# Predictors of Treatment Failure in the Surgical Management of Peritonsillar Abscesses

**DOI:** 10.3390/jcm15218207

**Published:** 2026-10-22

**Authors:** Selene Miccichè, Maddalena Fontana, Simona Rusignuolo, Francesco Dispenza, Francesco Lorusso, Salvatore Gallina, Angelo Immordino, Federico Sireci

**Affiliations:** 1 Unit of Otorhinolaryngology, Department of Biomedicine, Neuroscience and Advanced Diagnostics, Azienda Ospedaliera Universitaria Policlinico “Paolo Giaccone”, University of Palermo, Via del Vespro, 133, 90127 Palermo, Italy; selenemicciche@gmail.com (S.M.); maddalena.fon@gmail.com (M.F.); francesco.dispenza@unipa.it (F.D.); angelo.immordino182@gmail.com (A.I.); 2 Otorhinolaryngology Section, Department of Precision Medicine in Medical, Surgical and Critical Care (Me.Pre.C.C), University of Palermo, 90133 Palermo, Italy

**Keywords:** peritonsillar abscess, surgical drainage, treatment failure, peritonsillar abscess with a concurrent intratonsillar abscess

## Abstract

**Objectives**: Peritonsillar abscess (PTA) is the most common deep neck infection and is usually managed with antibiotic therapy and surgical drainage. However, drainage may fail, leading to persistent disease, repeat intervention, tonsillectomy, or early recurrence. This study aimed to identify factors associated with drainage failure. **Methods**: We conducted a retrospective single-centre study of patients admitted for PTA between 2019 and 2024. All patients underwent antibiotic therapy and surgical drainage by needle aspiration or incision and drainage. Drainage failure was defined as absence of pus evacuation, lack of clinical improvement within 72 h, need for quinsy tonsillectomy (QT), or recurrence within 30 days. Clinical and radiological variables and drainage techniques were analysed using univariate and multivariable analyses. **Results**: Eighty-four patients were included; drainage was successful in 68 (81%) and failed in 16 (19%). Previous ipsilateral PTA, smaller abscess diameter, multiloculation, and peritonsillar abscess with a concurrent intratonsillar abscess were significantly associated with failure. Needle aspiration showed a higher failure rate than incision and drainage (33.3% vs. 12.3%; OR 3.57; 95% CI 1.16–11.00; *p* = 0.035), but was not significant after adjustment (OR 2.87; 95% CI 0.63–14.04; *p* = 0.170). Peritonsillar abscess with a concurrent intratonsillar abscess (OR 57.30; 95% CI 2.37–9371.97; *p* = 0.011) and previous ipsilateral PTA (OR 20.68; 95% CI 4.77–108.22; *p* < 0.001) were independent predictors of failure. **Conclusions**: Peritonsillar abscess with a concurrent intratonsillar abscess and previous ipsilateral PTA were independently associated with drainage failure. These findings may help identify patients at increased risk of unsuccessful drainage and support individualized management.

## 1. Introduction

Peritonsillar abscess (PTA) represents the most frequent suppurative complication of acute tonsillitis, as well as the most common abscess in the head and neck region. The estimated annual incidence in Europe is 19–37 cases per 100.000 inhabitants. Streptococcus pyogenes, other β-haemolytic streptococci (groups C and G), and obligate anaerobes play a predominant role in its aetiology. Mixed infections with aerobic and anaerobic bacteria are not uncommon [1].

The pathophysiology of PTA can be explained by two distinct theories. According to the first hypothesis, it develops from peritonsillar cellulitis, which progresses into a localized purulent collection in the peritonsillar space. The second theory considers PTA as the consequence of abscess formation in a group of salivary glands (Weber’s glands) located in the peritonsillar space [2].

Clinical presentation of PTA is characterized by fever, general malaise, sore throat, odynophagia, referred otalgia, muffled (“hot potato”) voice, dysphagia, and drooling. Upon oropharyngeal examination, which may be limited by trismus, the typical objective findings include medial displacement of the palatine tonsil and hyperaemia and swelling of the ipsilateral soft palate. Painful lateral cervical lymphadenopathy is often present.

Diagnosis is mainly clinical. Imaging is not routinely required, but it is useful in selected cases: doubtful diagnosis, suspected extension to the deep neck spaces, airway compromise, or failure of abscess drainage [3,4]. The contrast-enhanced CT shows the abscess collection as a hypodense area in the peritonsillar space, with ring enhancement and is often associated with surrounding tissue edema.

The treatment is based on broad-spectrum empirical antibiotic therapy, which may be associated with surgical drainage of the abscess by needle aspiration, incision, or quinsy tonsillectomy (QT). Currently available guidelines indicate surgical drainage of the abscess as a fundamental component of treatment, supporting the fact that a combined medical and surgical approach leads to faster resolution of the clinical presentation and reduces the possibility of abscess extension and recurrence [5].

However, surgical drainage of the abscess is not always effective in achieving pus evacuation from the peritonsillar space. The literature reports a surgical drainage failure rate ranging from 4% to 15.5% [6,7]. The aim of this study was to evaluate the clinical–radiological factors and surgical techniques associated with a higher failure rate of surgical drainage of PTA, in order to identify predictors of unfavorable outcome and optimize therapeutic management, thus contributing to evidence-based clinical practice and better individualized therapeutic planning.

## 2. Materials and Methods

We conducted a single-centre retrospective study of patients admitted with PTA to the Department of Otorhinolaryngology, University Hospital of Palermo, between 2019 and 2024. Patients were included based on the presence of ICD-9 code 475 “peritonsillar abscess” in discharge records.

Medical records and imaging studies were reviewed.

Patients with concomitant neck abscesses treated with medical therapy alone were excluded from the study.

**Figure 1:**
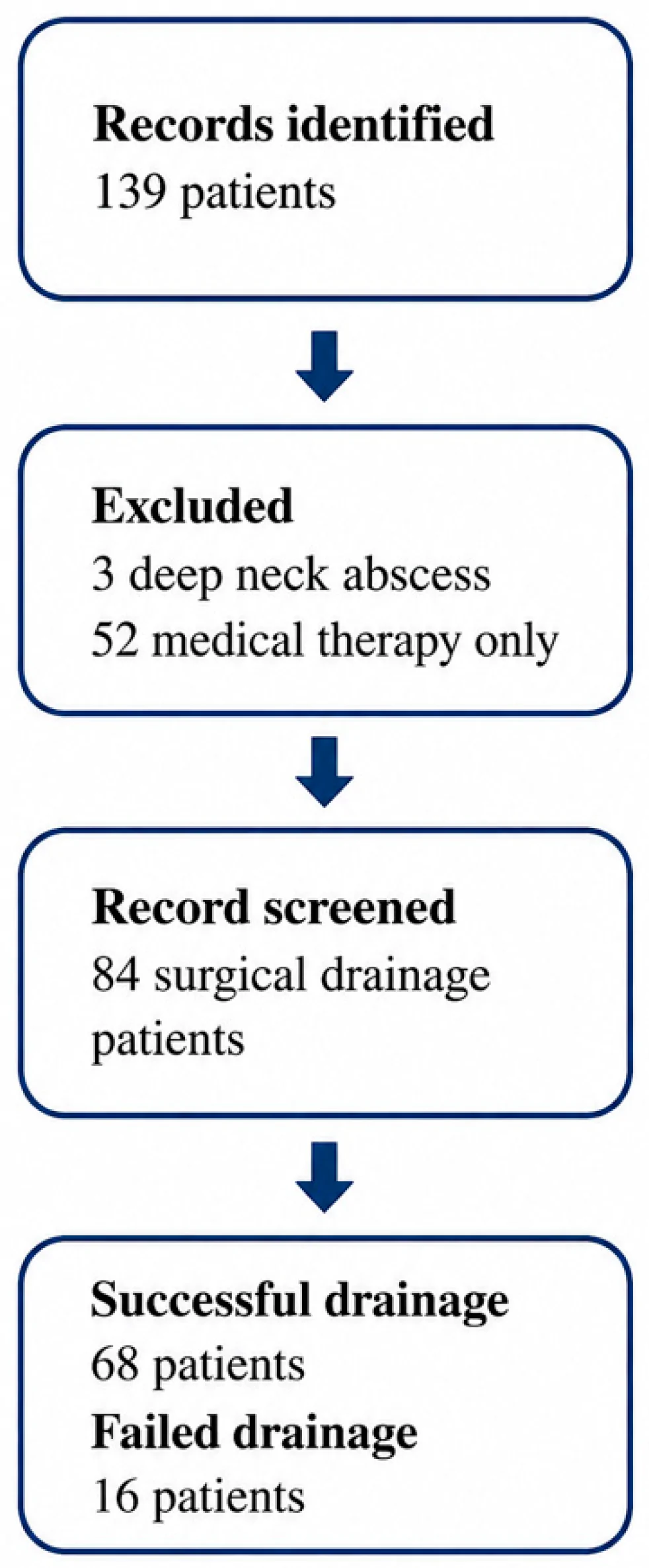
Flowchart of patient selection.

The study patients were divided into two groups: patients treated with surgical drainage of the PTA with procedural success (Group A) and patients treated with surgical drainage of the PTA with procedural failure (Group B), as shown in the flowchart in Figure 1.

Drainage failure was defined as absent pus drainage, no clinical improvement within 72 h, the need for QT, or recurrence within 30 days.

Collected variables included sex, age, previous PTA, symptoms, clinical signs, and radiological findings. All patients included in the present cohort had undergone contrast-enhanced CT as part of the local emergency department diagnostic pathway during the study period. This reflects local practice and should not be interpreted as an indication for routine CT imaging in otherwise uncomplicated PTA. Radiological characteristics of the abscess evaluated included size, location, and multiloculation. Regarding localization, the presence of involvement exclusively in the peritonsillar region or extension into the intratonsillar region was evaluated. Peritonsillar abscess with a concurrent intratonsillar abscess was defined as radiological evidence of extension of the peritonsillar collection into the tonsillar parenchyma.

All patients underwent drainage under local anaesthesia (10% lidocaine spray). Incision was made at the point of maximal swelling of the anterior tonsillar pillar. The two surgical techniques used were needle aspiration and incision and drainage. The incision was performed with a fine-tipped Klemmer or scalpel and gently dilated to facilitate pus drainage.

As no standardized protocol was available, the choice between needle aspiration and incision was left to the attending surgeon based on abscess characteristics, patient tolerance and anatomical accessibility. Needle aspiration was generally preferred for upper-pole abscesses with good visualization and patient compliance, whereas incision was favoured in more lateral collections or in cases of limited exposure.

Patients with persistent or worsening symptoms within 72 h after surgical drainage underwent QT during hospitalization. Elective tonsillectomy was recommended after recovery in all other cases.

Groups were compared using Student’s t-test for continuous variables and Fisher’s exact test for categorical variables. Given the limited number of drainage failure events (*n* = 16) and the presence of complete separation for peritonsillar abscess with a concurrent intratonsillar abscess, multivariable analysis was performed using Firth’s penalized logistic regression to reduce small-sample bias and provide finite parameter estimates. The model included three clinically relevant variables: peritonsillar abscess with a concurrent intratonsillar abscess, previous ipsilateral PTA, and surgical technique. Results were expressed as odds ratios (ORs) with 95% confidence intervals (95% CIs). A *p* value < 0.05 was considered statistically significant. Statistical analyses were performed using SPSS 25.0 (R Foundation for Statistical Computing, Vienna, Austria).

The study was conducted in accordance with the Declaration of Helsinki and was approved by the Ethics Committee of the University Hospital of Palermo. Given the retrospective nature of the study, the requirement for informed consent was waived.

Generative artificial intelligence (ChatGPT-6) was used to assist in the creation and graphical design of the graphical abstract and selected figures.

## 3. Results

Between 2019 and 2024, 139 patients were admitted with PTA. Three patients with concomitant neck abscesses (2 parapharyngeal abscesses, 1 retropharyngeal abscess) and 52 patients treated with medical therapy alone were excluded, leaving 84 patients for analysis.

The cohort included 47 males (56%) and 37 females (44%), with a mean age of 36.22 ± 14.8 years.

Drainage was successful in 68 patients (81%) and failed in 16 (19%).

**Figure 2:**
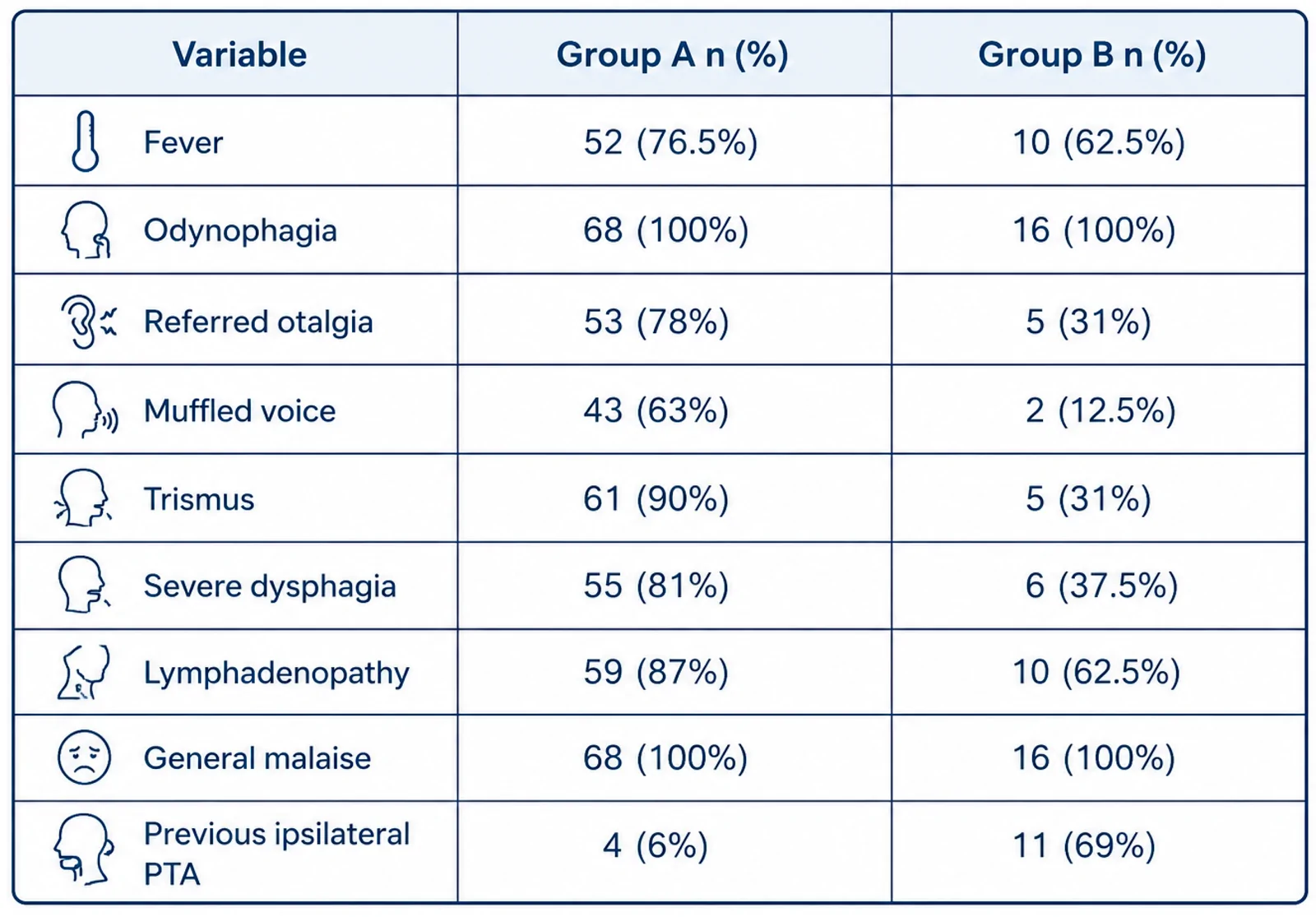
Comparison of clinical characteristics between patients with successful (Group A) and failed (Group B) surgical drainage of PTA.

Clinical characteristics at hospital admission are summarized in Figure 2.

Trismus was more frequent in Group A than in Group B (90% vs. 31%; OR 19.17; 95% CI 5.15–71.41; *p* < 0.0006). Muffled voice (63% vs. 12.5%; OR 12; 95% CI 2.53–57.38; *p* = 0.0005), referred otalgia (78% vs. 31%; OR 7.77; 95% CI 2.34–25.87; *p* < 0.0006), and severe dysphagia (81% vs. 37.5%; OR 7.05; 95% CI 2.17–22.92; *p* = 0.0011) were also more frequent in Group A. No significant differences were found for fever (76.5% vs. 62.5%; *p* = ns) and lateral cervical lymphadenopathy (87% vs. 62.5%; *p* = ns). Sore throat, odynophagia, and general malaise were present in all patients in both groups.

A positive history of previous ipsilateral PTA was significantly more common in Group B than in Group A (69% vs. 6%; OR 35.2; 95% CI 8.16–151.90; *p* < 0.0001).

Regarding CT findings, in Group A the mean maximum abscess diameter was 31 ± 12.5 mm.

In 11 patients (16%), the abscess was multiloculated and none showed peritonsillar abscess with a concurrent intratonsillar abscess, remaining confined to the peritonsillar space.

In Group B, the mean maximum abscess diameter was 22.1 ± 8.6 mm.

Multiloculation was present in 7 patients (44%) and peritonsillar abscess with a concurrent intratonsillar abscess in 5 patients (31%).

Radiologically, abscess diameter was significantly greater in Group A than in Group B (mean 31 ± 12.5 mm vs. 22.1 ± 8.6 mm; t(28.4) = 2.95; *p* = 0.006). Multiloculated collections were more frequent in Group B (44% vs. 16%; OR 4.03; 95% CI 1.24–13.12; *p* = 0.035). Peritonsillar abscess with a concurrent intratonsillar abscess occurred only in Group B (31% vs. 0%; *p* = 0.00014).

Regarding the surgical technique used, needle aspiration was performed in 18/68 patients (26.5%) in Group A and in 9/16 (56.3%) in Group B, whereas incision and drainage was performed in 50/68 (73.5%) and 7/16 (43.8%), respectively. Overall, treatment failure occurred in 9/27 patients (33.3%) managed with needle aspiration versus 7/57 (12.3%) managed with incision and drainage.

In Group B, 5/16 patients (31.3%) experienced worsening or no improvement within 72 h after drainage and clinical resolution was achieved only after QT. CT imaging of these 5 patients showed peritonsillar abscess with a concurrent intratonsillar abscess; in 3/5 patients, the initial drainage attempt under local anaesthesia did not show purulent discharge. Nine patients in Group B (9/16, 56.3%) showed clinical improvement despite the absence of purulent discharge during the procedure, suggesting improvement with medical therapy alone. Finally, 3/16 patients (18.8%) had purulent discharge and initial clinical improvement but presented an early recurrence within 30 days (Figure 2).

These patients were readmitted to our department, where they underwent a further attempt at drainage of the PTA, with success of the surgical procedure and clinical healing.

For univariate analysis, needle aspiration was associated with a higher failure rate than incision and drainage (33% vs. 12%; OR 3.57; 95% CI 1.16–11.00; *p* = 0.035). Multiloculation was also significantly associated with drainage failure on univariate analysis (44% vs. 16%; OR 4.03; 95% CI 1.24–13.12; *p* = 0.035).

In the multivariable analysis performed using Firth’s penalized logistic regression, peritonsillar abscess with a concurrent intratonsillar abscess (adjusted OR 57.30; 95% CI 2.37–9371.97; *p* = 0.011) and previous ipsilateral PTA (adjusted OR 20.68; 95% CI 4.77–108.22; *p* < 0.001) were independently associated with drainage failure. Needle aspiration was associated with increased odds of failure but did not retain statistical significance after adjustment (adjusted OR 2.87; 95% CI 0.63–14.04; *p* = 0.170).

## 4. Discussion

The present study identified peritonsillar abscess with a concurrent intratonsillar abscess and a history of previous ipsilateral PTA as the factors independently associated with drainage failure. Smaller abscess size and multiloculation were also associated with failure in univariate analysis, while needle aspiration showed a higher failure rate than incision and drainage, although this association was not maintained after multivariable adjustment. Overall, drainage was successful in 81% of patients, whereas treatment failure occurred in 19%.

The drainage failure rate observed in our cohort (19%) was higher than that reported in the literature (4% to 15.5%) [6,7]. This discrepancy may depend on differences in failure definitions.

Johnson et al. [6] defined failure as lack of symptom resolution, need for repeat drainage, or the performance of QT. Our definition partially overlaps but is broader, including absence of pus drainage and recurrence within 30 days.

Agerhäll et al. [7] primarily defined failure as recurrence within 30 days or the need for QT. Our definition of drainage failure was intentionally comprehensive, incorporating technical, clinical, and early recurrence criteria to capture incomplete resolution rather than new episodes. Importantly, however, absence of pus evacuation should not necessarily be interpreted as technical drainage failure, as it may also reflect a non-drainable or incompletely organized inflammatory collection. In particular, inflammatory or phlegmonous changes may mimic a small abscess on contrast-enhanced CT, potentially leading to false-positive radiological findings. This possibility should be considered when interpreting cases in which no purulent material is obtained despite CT findings suggestive of an abscess. Clinical presentation differed significantly between patients in Group A (successful drainage) and Group B (failed drainage). Patients in Group A more frequently presented classic symptoms of advanced PTA (trismus, referred otalgia, muffled voice, severe dysphagia). These symptoms are suggestive of an advanced infection, with significant involvement of peritonsillar tissues, which often corresponds to a well-formed abscess cavity, generally easier to drain.

In contrast, patients in Group B less frequently showed these symptoms, suggesting that in these cases the collections might have been incompletely organized or more difficult to access because of their small size. Notably, nine patients classified as drainage failures showed clinical improvement despite the absence of purulent discharge during the procedure. This finding raises the possibility that some CT-detected collections represented incompletely organized abscesses or phlegmonous inflammatory changes rather than freely drainable purulent collections. Although previous studies and clinical recommendations suggest that these symptoms are indicators to opt for surgical drainage, no publications have clearly linked them to procedural success. Galioto et al. [8] reported that for small PTA (<1 cm) without trismus and muffled voice, medical therapy alone may be sufficient for disease resolution. Our results are consistent with this indication: the absence of typical symptoms of more mature abscesses was more frequent in the group in which drainage was unsuccessful. Thus, in selected patients, medical therapy with close follow-up may be appropriate.

No differences were found for fever, lateral cervical lymphadenopathy, or general malaise, indicating that these systemic signs are not predictive of drainage outcome. They likely reflect a nonspecific inflammatory response and do not correlate with abscess maturity or size.

A particularly relevant finding was the association between previous recurrence and drainage failure, confirmed by multivariate analysis. In our cohort, 69% of patients in Group B reported at least one previous ipsilateral PTA episode versus 6% in Group A, suggesting that recurrence history is an independent predictive factor of unsuccessful drainage. To our knowledge, this association has not been specifically evaluated in previous studies. From a pathophysiological perspective, repeated inflammatory episodes may induce structural changes in the tonsillar and peritonsillar tissues, including fibrosis and pericapsular sclerosis [9]. Such alterations could potentially distort local anatomy and may contribute to the development of anatomically complex collections, thereby making complete drainage more difficult.

From a pathophysiological perspective, repeated infections may induce permanent local changes, such as fibrosis, adhesions, distortion of fascial planes, and formation of irregular or multiloculated cavities. These phenomena may alter the normal architecture of the peritonsillar space, favouring atypical collections, such as a peritonsillar abscess with a concurrent intratonsillar abscess or internal septa that make complete drainage under local anaesthesia difficult.

Imaging findings showed that abscesses with a greater maximum diameter were significantly more frequent in Group A than in Group B. This finding is fully consistent with the most recent literature, which suggests that the size of the collection is an indicator of the success of surgical drainage.

Li et al. [10] demonstrated that the maximum diameter of the abscess was greater in cases with effective drainage, with mean values of approximately 26 mm in successfully drained cases compared with 20 mm in those with drainage failure. These data suggest that abscess size correlates with the outcome of surgical drainage and that smaller collections are more frequently associated with failure.

Therefore, in these cases, a conservative management approach may be more appropriate before performing surgical drainage. Indeed, several studies and guidelines suggest that for small abscess collections, conservative management based solely on medical therapy may be appropriate [8,11,12].

In our analysis, multiloculation was more frequent in Group B and was associated with drainage failure on univariate analysis, suggesting that multiloculated abscesses may be more difficult to drain completely. Multiloculated morphology has been previously described on CT in patients with PTA [13]; however, evidence specifically addressing its impact on drainage outcomes remains limited. From a technical perspective, the presence of multiple locules may hinder complete evacuation through a single drainage site. Our findings suggest that multiloculation may represent a radiological marker of difficult drainage, although this association requires confirmation in larger prospective studies.

Another highly relevant finding was that peritonsillar abscess with a concurrent intratonsillar abscess was observed exclusively in Group B (*n* = 5), with all cases requiring QT for resolution.

Peritonsillar abscess with a concurrent intratonsillar abscess represents a clinically relevant variant, in which the abscess involves the tonsillar parenchyma, an area not accessible to standard peritonsillar aspiration or incision, thereby limiting drainage efficacy.

The literature devotes specific attention to intratonsillar abscess, considering it a rarer nosological entity compared with peritonsillar abscess and one that often requires different management strategies. In cases of pure intratonsillar abscess, medical therapy alone is often effective. In refractory cases, targeted intraparenchymal aspiration, incision under general anaesthesia, or tonsillectomy may be useful strategies [14,15].

The cases described in our series were not pure intratonsillar abscesses but peritonsillar abscesses with a concurrent intratonsillar abscess. Our data highlight that peritonsillar abscess with a concurrent intratonsillar abscess represents a critical factor in PTA management. The presence of pus within the tonsillar parenchyma can explain the failure of traditional peritonsillar drainage, as the collection is not fully accessible externally.

From a therapeutic perspective, our experience suggests that QT represents a definitive approach in cases in which conventional drainage fails.

This is consistent with the literature, where QT is considered indicated in patients with intratonsillar abscess who do not respond to medical therapy. Moreover, the distinction between pure intratonsillar abscess and peritonsillar abscess with intraparenchymal extension is fundamental: in the former, antibiotic therapy may be sufficient, whereas in the latter, surgery is often unavoidable.

In our sample, needle aspiration was associated with higher failure rates than incision in univariate analysis (33% vs. 12%), but this association was not significant in multivariate analysis. This finding suggests that the apparent effect of drainage technique may be influenced by other clinical or anatomical factors.

The literature reports heterogeneous results on this topic. Some systematic reviews comparing needle aspiration with incision and drainage conclude that no clear consensus exists on which technique is superior [16,17].

Other studies highlight limitations of needle aspiration compared with incision, suggesting that, although both techniques are widely used, incision may reduce recurrence or the need for repeat procedures [18,19].

However, technique selection cannot be uniform and should consider multiple factors, including abscess anatomy (cases with peritonsillar abscess with a concurrent intratonsillar abscess or complex loculations seem less amenable to complete aspiration), patient comfort (aspiration may be less painful and therefore better tolerated), and clinical context (aspiration is technically simpler, faster, and less invasive).

Finally, an important clinical finding emerged from our data: in all five patients with CT-confirmed peritonsillar abscess with a concurrent intratonsillar abscess, drainage failed and resolution was achieved only through QT. This finding is consistent with previous studies showing substantially lower drainage success rates in intratonsillar abscesses compared with conventional PTA [14,20]. However, our cases represented peritonsillar abscesses with a concurrent intratonsillar abscess rather than pure intratonsillar abscesses; therefore, this comparison should be interpreted with caution.

QT represents a therapeutic option in selected cases, although it is not universally adopted as a first-line approach. A systematic review and meta-analysis by Tsikopoulos et al. found no significant difference in postoperative haemorrhage between immediate and interval abscess tonsillectomy, while interval tonsillectomy was associated with a longer hospital stay [21]. The authors concluded that immediate abscess tonsillectomy appears to be a safe therapeutic option, allowing complete abscess drainage and rapid symptom relief [21]. Accordingly, QT may be considered in selected clinical scenarios, particularly following failure of conventional drainage or when tonsillectomy is otherwise clinically indicated. Our findings are consistent with this approach, as all patients with peritonsillar abscess with a concurrent intratonsillar abscess in our cohort experienced drainage failure and subsequently underwent QT.

Elective tonsillectomy may also be considered after resolution of the acute episode in appropriately selected patients with recurrent PTA or recurrent tonsillitis, according to the individual clinical history and recurrence risk [22].

This study has several limitations. First, its retrospective, single-centre design may introduce selection bias and limits the generalizability of our findings. Second, the relatively small sample size and, particularly, the limited number of drainage failure events reduced the statistical power and constrained the robustness of the multivariable analysis. In addition, the drainage technique was not standardized and was selected according to clinical presentation and operator preference, potentially introducing treatment-selection bias. Operator experience, antibiotic regimen, and timing of drainage were also not standardized or fully controlled for and may have acted as residual confounders. Furthermore, relevant comorbidities, including diabetes and immunocompromised conditions, were not systematically collected and therefore could not be evaluated as potential factors associated with drainage failure. Finally, only a single drainage attempt was considered before treatment failure was assessed, which may have contributed to the observed failure rate.

## 5. Conclusions

In our retrospective study, surgical drainage of peritonsillar abscesses under local anaesthesia showed a failure rate of 19%. Peritonsillar abscess with a concurrent intratonsillar abscess and a history of previous ipsilateral PTA were independently associated with drainage failure. These findings suggest that anatomical characteristics of the collection and previous disease history may help identify patients at increased risk of unsuccessful conventional drainage. Further studies are warranted to confirm whether peritonsillar abscess with a concurrent intratonsillar abscess and previous ipsilateral PTA can reliably identify patients at increased risk of unsuccessful conventional drainage.

## Data Availability

The data presented in this study are available on reasonable request from the corresponding author. The data are not publicly available due to privacy restrictions related to patient confidentiality.
